# Supplementary figures and images for: FBXO11 governs macrophage cell death and inflammation in response to bacterial toxins
Source: Life Sci Alliance. 2023 Mar 28;6(6):e202201735. doi: 10.26508/lsa.202201735 (PMC10053445; doi:10.26508/lsa.202201735)

Figure 1E  
[Uncropped]

FBXO11

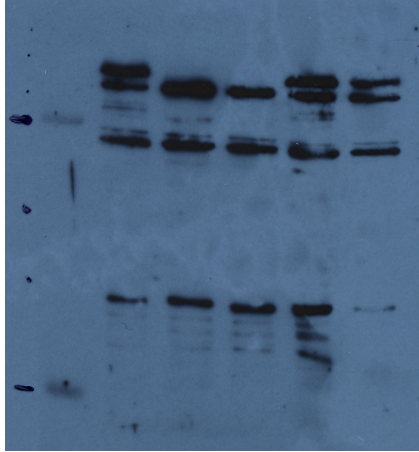

FBXO11 (longer exposure)

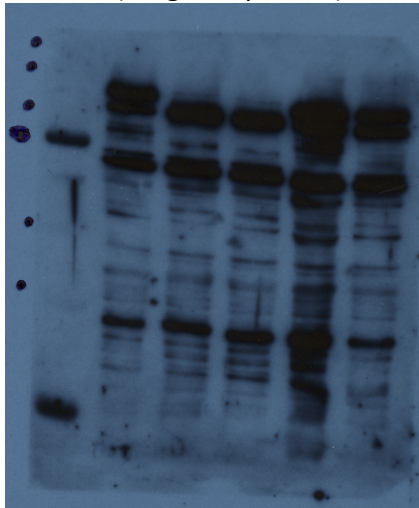

$\alpha$ -tubulin

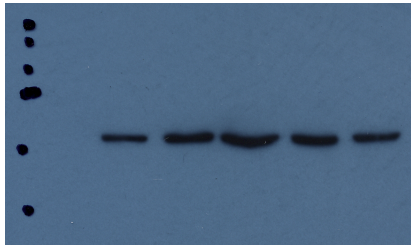

Figure 1F  
[Uncropped]

WT

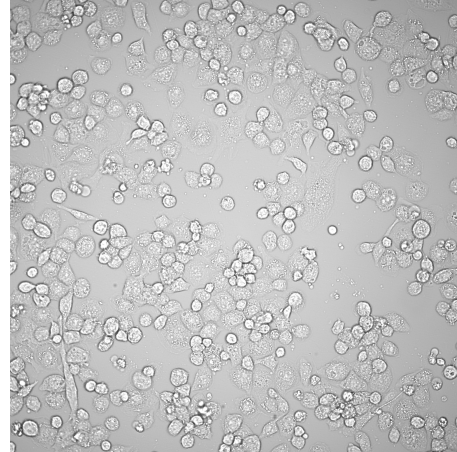

E2

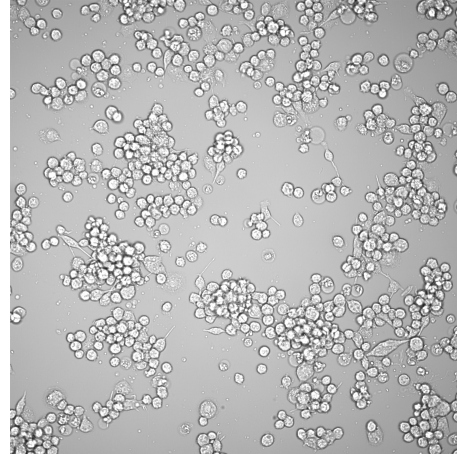

E3

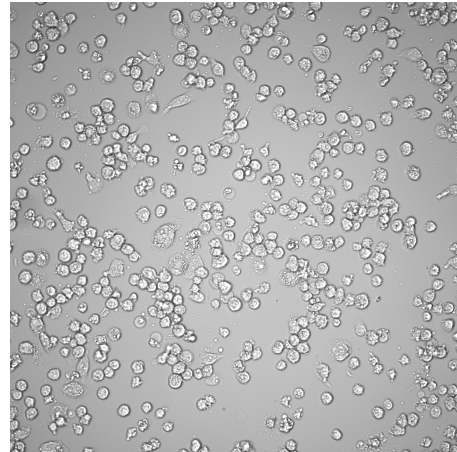

Supplement: Supplementary file 1 [file LSA-2022-01735_SdataF1.pdf]

Figure 2E  
[Uncropped]

C5aR1

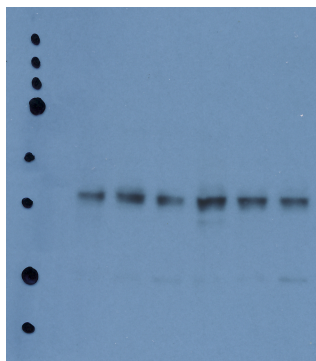

$\beta$ -actin (top bands)

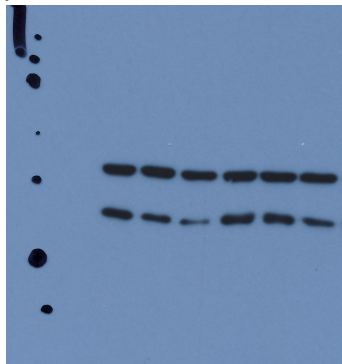

Supplement: Supplementary file 4 [file LSA-2022-01735_SdataF2.pdf]

Figure 3D  
[Uncropped]

C5aR1

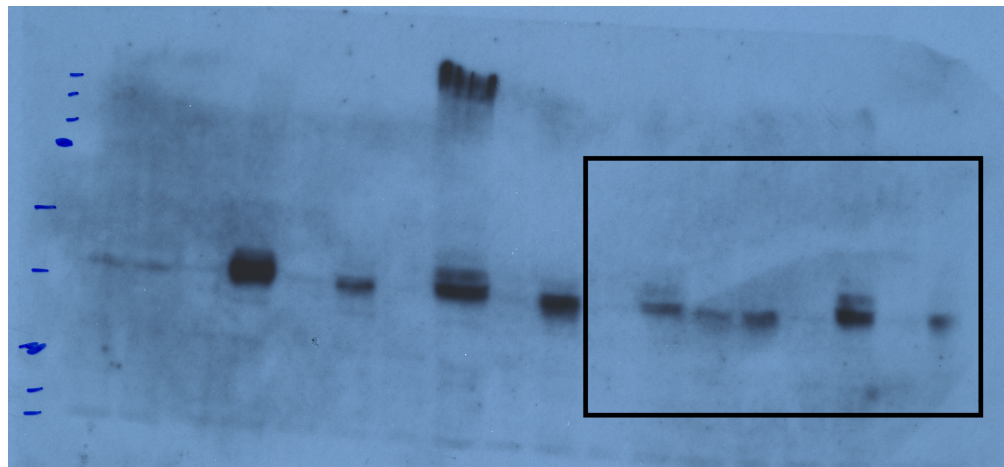

$\beta$ -actin

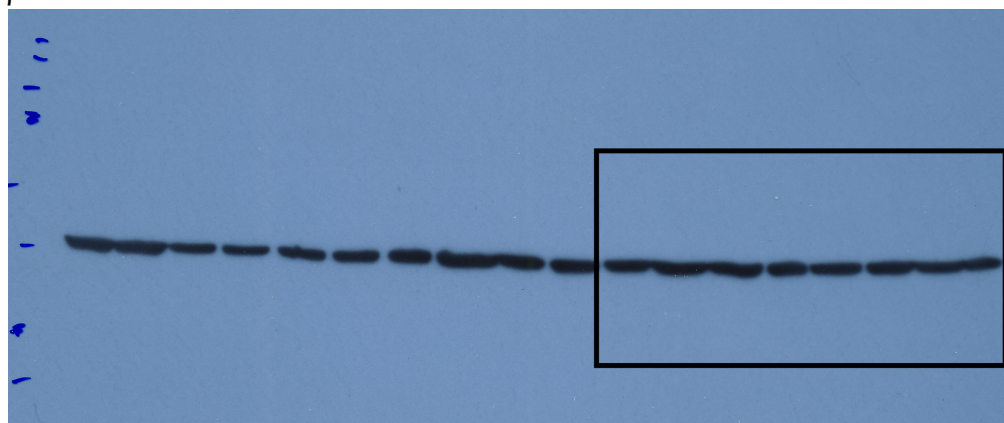

Supplement: Supplementary file 5 [file LSA-2022-01735_SdataF3.pdf]

Figure 4B  
[Uncropped]

C5aR1 (shorter and longer exposure)

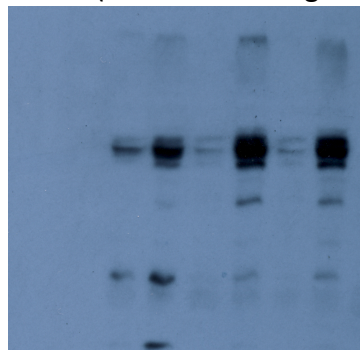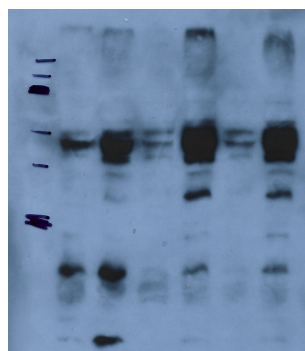

$\beta$ -actin

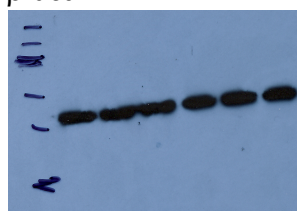

Supplement: Supplementary file 6 [file LSA-2022-01735_SdataF4.pdf]

Figure S2  
[Uncropped]

FBXO11 (top band)  
 $\alpha$ -tubulin (bottom band)

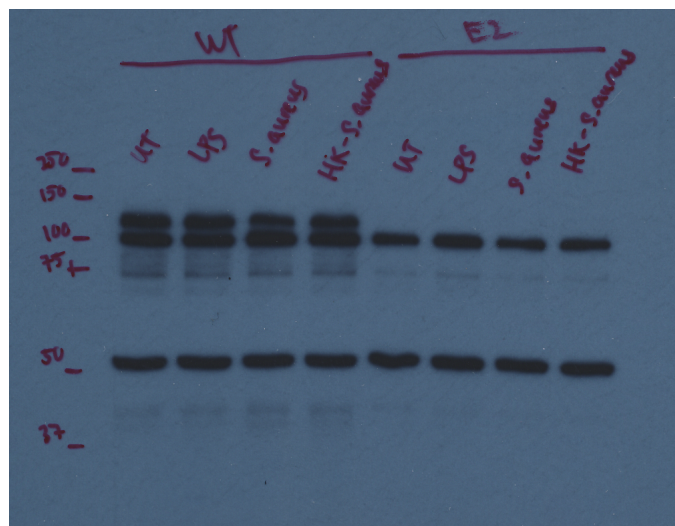

C5aR1

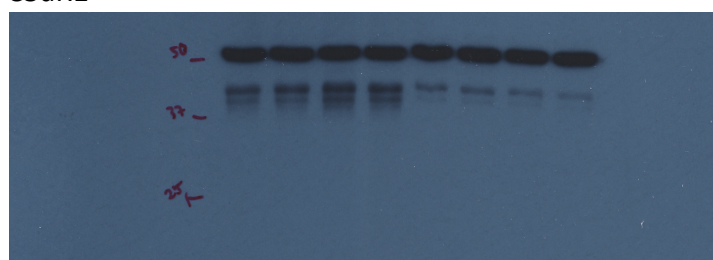

Supplement: Supplementary file 7 [file LSA-2022-01735_SdataFS3.pdf]

Figure 6A  
[Uncropped]

BCL6

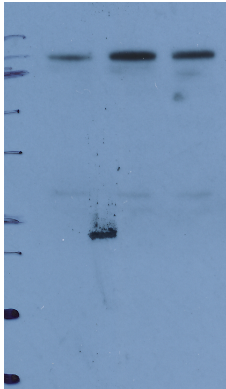

CD40

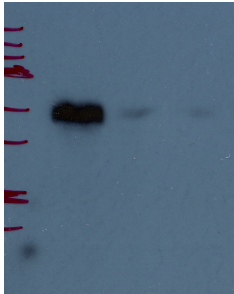

$\alpha$ -tubulin

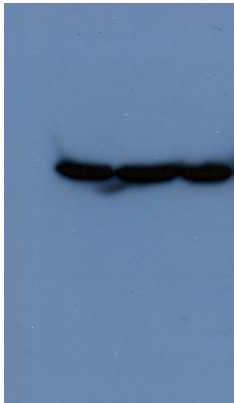

Figure 6D

BCL6

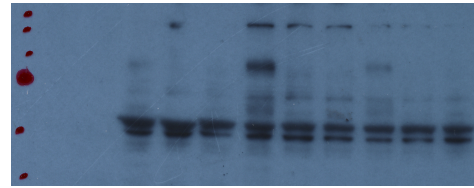

CD40

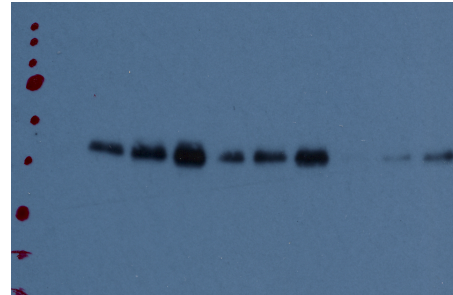

C5aR1

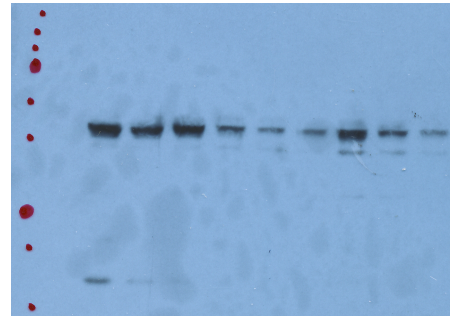

IL-1 $\beta$

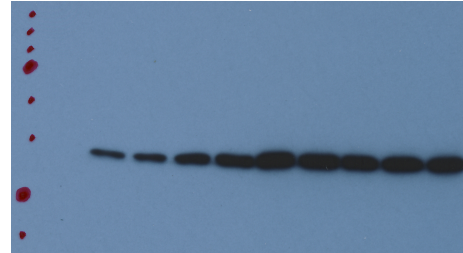

$\alpha$ -tubulin

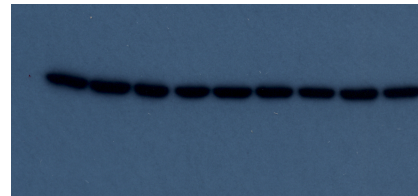

Supplement: Supplementary file 9 [file LSA-2022-01735_SdataF6.pdf]
