# Supplementary material for: FBXO11 governs macrophage cell death and inflammation in response to bacterial toxins
Source: Life Sci Alliance. 2023 Mar 28;6(6):e202201735. doi: 10.26508/lsa.202201735 (PMC10053445; doi:10.26508/lsa.202201735)

Figure 5C
[Uncropped]

IL-1β (lysate)

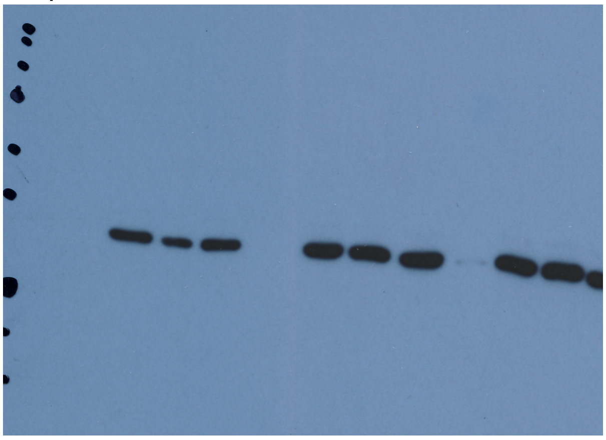

β-actin

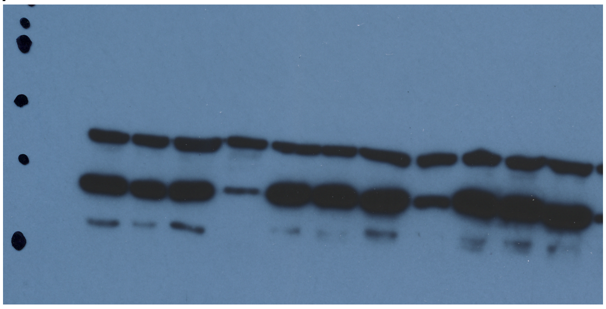

IL-1β (supernatant – long exposure)

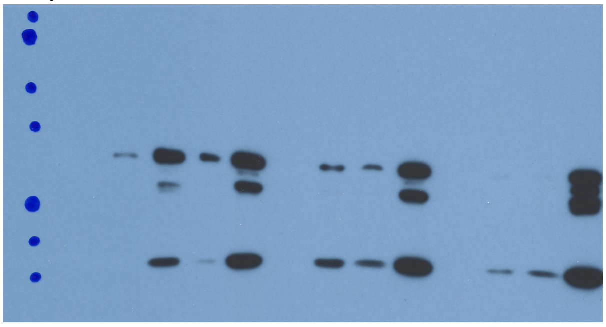

IL-1β (supernatant – short exposure)

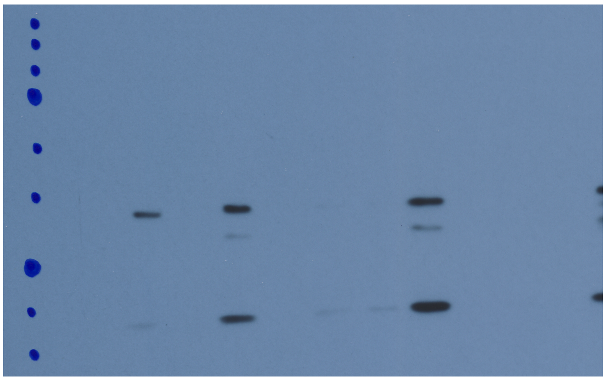

Figure 5D
[Uncropped]

IL-1β

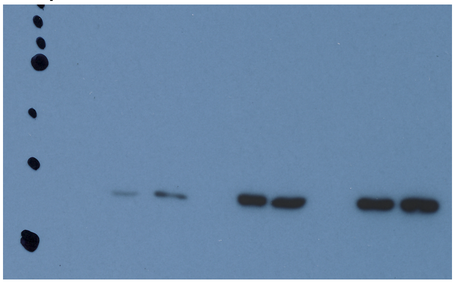

β-actin

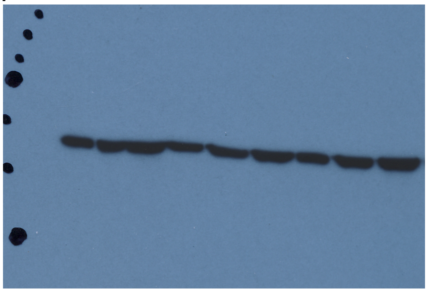

Figure 5E
[Uncropped]

NLRP3 (top band)

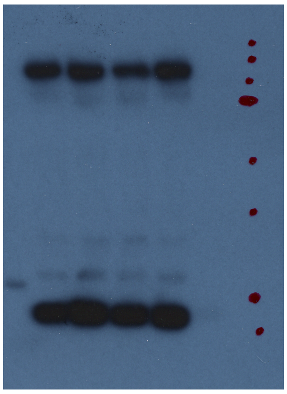

β-actin (bottom band)

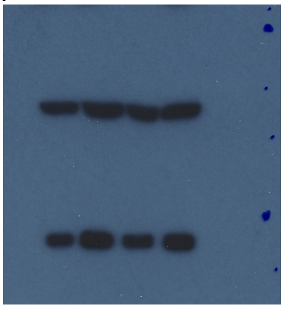

Figure 5F

IL-1β (short exposure)

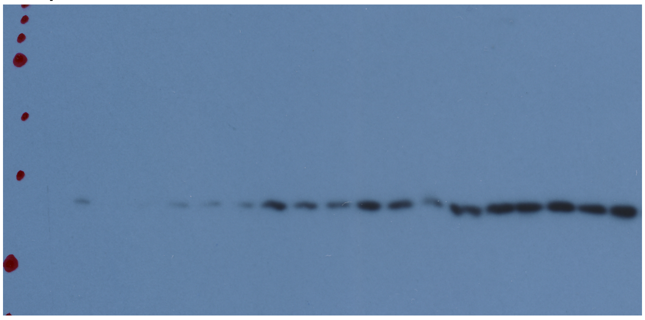

IL-1β (long exposure)

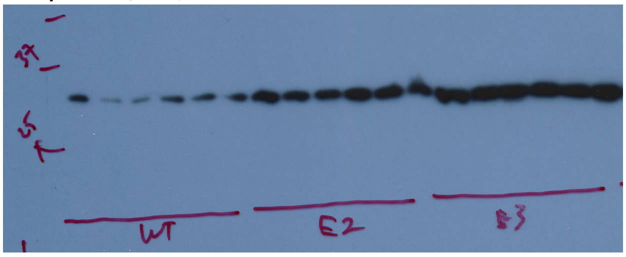

MCL-1

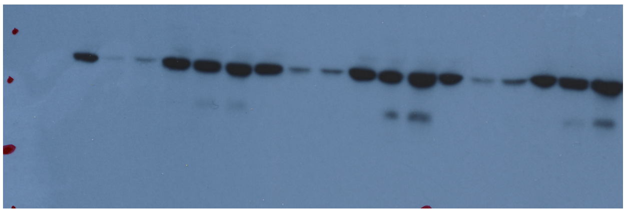

α-tubulin

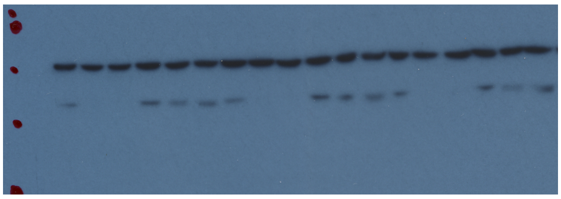

Figure 5G
[Uncropped]

IL-1β

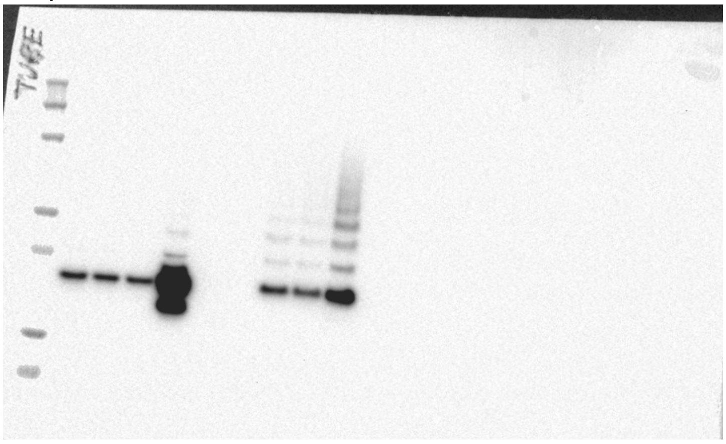

Ponceau

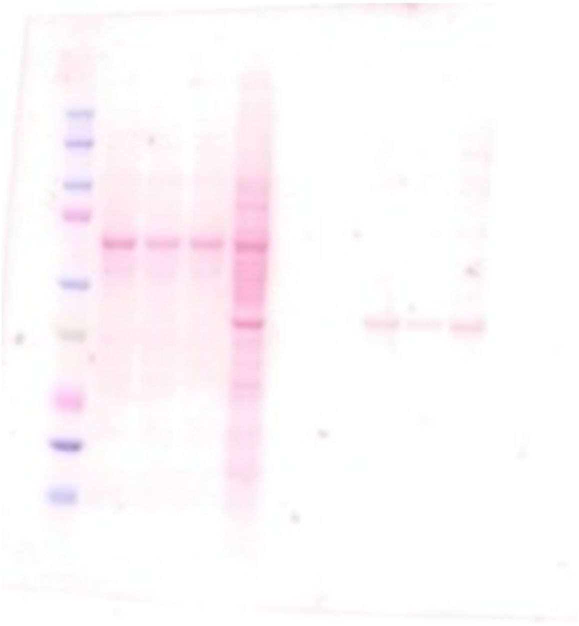

Supplement: Supplementary file 8 [file LSA-2022-01735_SdataF5.pdf]
